# Hospitals and Their Work in Mauritius

**Published:** 1905-12-23

**Authors:** 


### HOSPITALS AND THEIR WORK IN MAURITIUS.

In his report for 1904, just issued, Mr. D. C. Cameron, Acting Colonial Secretary for Mauritius, makes some interesting references to medical work and its difficulties in the Colony. There are twelve hospitals and twenty-eight dispensaries in the island besides Poor-law Infirmaries and a leper asylum. Forty-one lepers were admitted during 1904, and 104 remained in the asylum at the close of the year. The total number of admissions to the various hospitals for the year was 17,492.

The tropical climate of Mauritius appears to be answerable for the greater part of disease and mortality. The atmosphere is hot and damp. Earthquakes are of frequent occurrence, sixty-five shocks having been recorded by the seismograph at the Royal Alfred Observatory last year. The cyclones, accompanied by very heavy rains, leave the land in an unhealthy condition and encourage the distribution of disease by the rapid propagation of mosquitoes. The year under consideration showed a slight improvement over 1903, there being a diminution in the number of cases of malaria, influenza, and dysentery, while the prevalence of plague is, next to that of 1902, the lowest hitherto recorded.

The number of patients suffering from tuberculosis has also slightly decreased since 1903. There is little hope of any lasting improvement in the number of these cases under the existing conditions of widespread poverty, ignorance of sanitation, and inadequate accommodation for the poor. Racial and social prejudices, too, tend to aggravate these factors, for Mauritius contains a greater mixture of nationalities than perhaps any other place of its size and importance. Descendants of the original French inhabitants and British merchants and government officials form the chief elements of "Society," while the working community is largely composed of natives, Indians, Africans, and Creoles and a great number of Chinese. As an example of this the police force is recruited from ten nationalities, two thirds only being natives of Mauritius.

One satisfactory item in the report on the vital statistics is the low rate of infantile mortality, but to counterbalance this a very high proportion of still-births—namely, 8.5 per cent.—is recorded. This specially obtains among the Indian population, and it appears that a large number of births are unregistered owing to death intervening before registration is made. The large proportion of still-births is doubtless due in part to a lack of midwifery aid. But even were that forthcoming in sufficient amount, it would probably take considerable time and influence to overcome the prejudices of race and religion which are often strongest in the matter of birth and of death.

